# Isolation, Identification and Genomic Characterization of Carbamazepine-Tolerant Bacteria from Contaminated Aquatic Environments

**DOI:** 10.3390/microorganisms14102305

**Published:** 2026-10-10

**Authors:** Gabriela Méndez, Diana Carrera, Lizbeth Olivo Silva, Elena Coyago-Cruz, Melisa Ortiz, Ivonne Vega

**Affiliations:** 1 Carrera de Biotecnología, Universidad Politécnica Salesiana, Sede Quito, Campus El Girón, Av. 12 de Octubre N2422 y Wilson, Quito 170143, Ecuador; gmendez@ups.edu.ec (G.M.);; 2 Master Innovative Drugs Parcours Nanomedicine, Université Marie et Louis Pasteur (UMLP), Site Besançon, 1 rue Claude Goudimel, 25030 Besançon Cedex, France; 3 Independent Researcher, Quito 170517, Ecuador

**Keywords:** carbamazepine, bacterial tolerance, environmental bacteria, 16 S rRNA gene, whole-genome sequencing, *Raoultella ornithinolytica*, xenobiotic metabolism, aromatic-compound metabolism

## Abstract

Pharmaceutical contamination in aquatic environments has increased interest in microorganisms able to tolerating these compounds and the mechanisms underlying their tolerance. This study isolated and characterized carbamazepine (CBZ)-tolerant bacteria from contaminated waters of the Machángara River, Quito, Ecuador, and investigated the genome of the most tolerant isolate. Isolates were characterized by morphological and biochemical analyses, and 16S rRNA gene sequencing. Tolerance to CBZ (1–50 mg L^−1^) was evaluated by culture-based growth measurements. CBZ1, CBZ3, CBZ6, and CBZ7 were taxonomically assigned to *Enterobacter hormaechei*, *Paenibacillus lactis*, *Aeromonas hydrophila* and *Raoultella ornithinolytica*, respectively. Among the four isolates evaluated, CBZ7 exhibited significantly higher relative growth at elevated CBZ concentrations (30–50 mg L^−1^) and was therefore selected for whole-genome sequencing. The CBZ7 draft genome comprised 5,671,759 bp distributed across 181 contigs, with a G+C content of 55.65%, an N50 of 79,780 bp, 98.2% BUSCO completeness, and CheckM2 estimates 0.87% contamination. Genome annotation revealed genes associated with membrane transport and efflux, oxidative metabolism, glutathione metabolism, environmental sensing, and aromatic compound metabolism. Although no complete CBZ-specific catabolic pathway was identified, the results support strong tolerance of CBZ7 to elevated CBZ concentrations and identity candidate functions potentially involved in xenobiotic adaptation.

## 1. Introduction

The increasing presence of pharmaceutical compounds in aquatic environments has raised growing concern because conventional wastewater treatment systems do not completely remove many of these contaminants, including carbamazepine (CBZ), acetaminophen, diclofenac, and ibuprofen. Carbamazepine is commonly detected in treated wastewater and surface waters because of its continuous release, incomplete removal during wastewater treatment and relatively high environmental persistence [1]. As a chronically toxic compound, carbamazepine can cause endocrine disruption, promote antibiotic resistance in human pathogens, trigger severe hypersensitivity reactions, and pose teratogenic risks during pregnancy. These adverse effects have led to its classification as an emerging pollutant and highlight the need for effective treatment strategies [2].

Microorganisms are a promising option for the remediation of pharmaceutical-contaminated environments. Bacteria are particularly relevant because of their metabolic versatility and ability to tolerate xenobiotic stress, and previous studies have demonstrated the transformation of CBZ by some bacterial strains under laboratory conditions. Bacterial tolerance to toxic compounds can involve multiple mechanisms, including efflux of toxic compounds from the membrane compartment, together with metabolic and oxidative-stress responses. The expression and effectiveness of these mechanisms depend on environmental conditions that support microbial growth and enzymatic activity [1,3].

Despite increasing interest in microbial responses to pharmaceutical contaminants, limited information is available on bacteria naturally exposed to CBZ-contaminated aquatic environments, particularly regarding the genomic traits associated with tolerance. Environmental isolates from chronically contaminated sites may therefore represent a valuable source of microorganisms adapted to pharmaceutical stress and may provide insight into the genetic mechanisms underlying their survival under CBZ exposure.

Accordingly, this study aimed to isolate and characterize CBZ-tolerant bacteria from contaminated waters of the Machángara River in Quito, Ecuador, evaluate their tolerance to increasing CBZ concentrations, and characterize the genome of the isolate exhibiting the strongest tolerance to identify functions potentially associated with xenobiotic adaptation. The results identified strain CBZ7 as the isolate with the strongest tolerance profile at elevated CBZ concentrations, and revealed a genomic repertoire associated with membrane transport, efflux, oxidative metabolism, glutathione metabolisms, environmental sensing, and aromatic compound metabolism. However, no complete CBZ-specific catabolic pathway was identified.

## 2. Materials and Methods

### 2.1. Reagents and Culture Media

Carbamazepine (purity 99%) was purchased from Sigma Life Science, St Louis, MO, USA and used to prepare standard stock solutions. The growth medium employed in this study was Mineral Salt Medium (MSM), composed of (in g L^−1^): Na_2_HPO_4_·2H_2_O (3.5), KH_2_PO_4_ (1.0), (NH4)_2_SO_4_ (0.225), MgSO_4_·7H2O (0.5), CaSO_4_·2H_2_O (0.045) and agar (15) [4]. The medium was sterilized by autoclaving and supplemented with carbamazepine. Following autoclaving, 1 mL of trace elements solution was added under sterile conditions. The trace element solution contained (in g L^−1^): NaHCO_3_ (2.0), MnSO_4_·4H_2_O (0.3), ZnSO_4_·7H2O (0.2), (NH_4_)_6_Mo_7_O_24_·4H2O (0.02), CuSO_45_H_2_O (0.1), CaCl_2_ 2H_2_O (0.05), FeSO_4_·7H2O (0.5). All chemicals used for the preparation of the mineral basal medium (MBM) were purchased from different suppliers. The pH of MSM used for this experiment was adjusted around 7.2  ±  0.2 prior to sterilization.

### 2.2. Isolation and Screening of Indigenous Carbamazepine-Tolerant Bacteria

Carbamazepine-tolerant bacteria were isolated from the Machángara River (0°15′25.4″ S 78°31′33.8″ W), one of the most contaminated water bodies in Quito, Ecuador, according to Méndez et al. [5]. Water samples were collected in amber glass bottles (200 mL) and transported to the laboratory for bacterial isolation.

To isolate potential carbamazepine-tolerant bacterial strains, a 2% (*v*/*v*) river water sample was inoculated into a 250 mL flask containing 200 mL of Mineral Salt Medium (MSM) broth supplemented with 2 mg L^−1^ of carbamazepine (CBZ). The culture was incubated at 26 °C under shaking conditions (150 rpm) for 15 days to allow bacterial acclimation to CBZ [6,7].

Following acclimation, the enrichment culture was serially diluted from 10^−1^ to 10^−4^ using sterile distilled water. An aliquot of 1 mL of each dilution was inoculated into a sterile Petri dish and mixed with approximately 25 mL of mineral salt medium (MSM) agar supplemented with 2 mg L^−1^ of carbamazepine, using the pour-plate technique. The plates were incubated aerobically at 26 °C for 24 h in an OVFU OBIS-1518D incubator.

After incubation, colonies exhibiting distinct morphological characteristics were selected and streaked onto MSM agar supplemented with CBZ and Tryptic Soy Agar (TSA) for further purification. The bacterial isolates were labeled with the prefix “CBZ” to denote their association with carbamazepine enrichment. Purification was repeated at least twice to obtain homogeneous cultures. Selected isolates were transferred to cryovials containing 25% glycerol and stored at −80 °C for long-term preservation. Pure cultures were also stored at 4 °C for subsequent identification assays.

### 2.3. Determination of Maximum Tolerance Concentration of Carbamazepine-Resistant Bacteria

The maximum tolerance concentration (MTC) of the carbamazepine-tolerant bacterial isolate was assessed by inoculating the strain into MSM supplemented with increasing concentrations of carbamazepine, ranging from 1 to 50 mg L^−1^. Before each assay, bacterial suspensions were prepared from pure cultures and adjusted to approximately a 0.5 McFarland standard. The optical density of each suspension was verified at 625 nm, and serial dilutions were subsequently prepared.

For each bacterial isolate and CBZ concentration (Sigma-Aldrich, St Louis, MO, USA), a 1 mL aliquot of the corresponding dilution was transferred to a sterile Petri dish and mixed with approximately 25 mL of MSM agar supplemented with the corresponding CBZ concentration using the pour-plate technique. The plate cultures were incubated at 26 °C in the dark.

As a growth reference control, the same bacterial inoculum and plated volume were evaluated on nutrient medium without CBZ. A sterility control containing MSM agar and the corresponding CBZ concentration without bacterial inoculum was also included. MSM without CBZ was evaluated separately as a negative medium control. All assays were performed in triplicate, and viable bacterial counts were expressed as colony-forming units per milliliter (CFU mL^−1^).

The absence of visible bacterial growth after the established incubation period was considered indicative of an inhibitory CBZ concentration. On the other hand, if visible growth was observed, it suggested that the tested carbamazepine concentration was not inhibitory. The MTC was defined as the highest CBZ concentration at which visible bacterial growth and countable colonies were observed.

To further evaluate bacterial tolerance, relative growth was calculated using the mean CFU mL^−1^ values obtained from the triplicates and the corresponding CBZ-free controls, according to Equation (1) [8].

(1) $$\%\;Relative\;growth\;\frac{Bacterial\;count\;in\;presence\;of\;carbamazepine\;(\frac{CFU}{mL})}{Bacterial\;count\;in\;nutrient\;medium\;control\;withouth\;carbamazepine\;(\frac{CFU}{mL})}\times 100$$

The numerator represents the viable bacterial count under CBZ exposure, whereas the denominator represents the viable count of the same isolate under nutrient-rich reference conditions without CBZ.

### 2.4. Biochemical Identification of Carbamazepine-Tolerant Bacterial Strains

The isolated carbamazepine-tolerant bacterial strains were selected and maintained as pure cultures on nutrient agar. Morphological characterization was carried out using Gram staining.

For biochemical identification, the oxidase test was performed using filter paper strips impregnated with tetramethyl-p-phenylenediamine dihydrochloride. The result was recorded within 60 s, considering a color change to blue or purple as a positive reaction. Subsequently, biochemical characterization was conducted using pure cultures after 24 h of incubation, applying the Microgen GnA + B − ID System. Reagents such as Kovac’s, VPI, VPII and TDA were added to the corresponding wells as specified in the manufacturer’s manual. Positive and negative results were determined based on observable color changes and using the Microgen ID System software (Version 1.2), which provided the identified microorganisms along with their percentage similarity to known species [9].

All results were compared with bacterial description in Bergey’s Manual of Determinative Bacteriology [10].

### 2.5. Molecular Identification and Phylogenetic Analysis Based on 16S rRNA Gene Sequencing

Genomic DNA was extracted from isolates with the highest percentage of tolerance using the Purelink Genomic DNA Mini kit (Thermo Fisher Scientific, Carlsbad, CA, USA), following the manufacturer’s protocol [11]. The purity and integrity of the extracted DNA were assessed by electrophoresis on a 1% agarose gel.

PCR amplification was performed in a Veriti^®^ 96-Well Thermal Cycler (Applied Biosystems, Foster City, CA, USA) under the following conditions: initial denaturation at 95 °C for 3 min, followed by another denaturation at 95 °C for 30 s, with a melting temperature of 56 °C for 30 s, an extension at 72 °C for 1.5 min and a final extension at 72 °C for 10 min; processes that were repeated for 35 cycles [12] with the universal primers (27 F) 5′-AGAGTTTGATCMTGGCTCAG3′ and (1492 R) 5′-TACCTTGTTACGATT-3′ [1]. DNA concentration was determined using Qubit 2.0 Fluorometer from Thermo Fisher Scientific, Whaltam, MA, USA. Amplified products were sent to MrDna Lab (Shallowater, TX, USA) and Macrogen Inc. Seoul, South Korea for Sanger sequencing.

The resulting raw sequences were cleaned in MEGA v12.0.7. Forward and reverse sequences were aligned to generate consensus sequences. These consensus sequences were compared against the GenBank database using NCBI BLASTN v2.16.0: https://blast.ncbi.nlm.nih.gov (accessed on 12 September 2025), and species identification was confirmed using the EZBioCloud: https://www.ezbiocloud.net/, (accessed on 12 September 2025) platform, allowing comparison with reference bacterial species [13]. Finally, the partial 16S rRNA gene sequences of the bacterial isolates were submitted to the GenBank database, and accession numbers were obtained for each bacterium strain (CBZ1: PV916048; CBZ3: PV916049, CBZ6: PV916050 and CBZ7: PV916051).

All obtained sequences were retrieved and subjected to multiple sequence alignment using the MUSCLE ClustalW algorithm [14]. To determine the taxonomic position of the isolate, a phylogenetic tree was constructed with MEGA v12.0.7. Evolutionary distances were calculated using the Tamura 3-parameter model [15]. Rare heterogeneity across sites was accounted for by applying a gamma distribution with a shape parameter of 5. A maximum likelihood tree was inferred to represent the evolutionary relationships among the sequences. The reliability of the tree topology was assessed through 1000 bootstrap replicates, and bootstrap support values were expressed as percentages at each node [16].

### 2.6. Whole-Genome Sequencing and Assembly

A paired-end DNA library was prepared by the external companies Macrogen Inc. (Seoul, Republic of Korea) and MrDna Lab (Shallowater, TX, USA), which performed the sequencing. Whole-genome sequencing was done on the Illumina MiSeq platform (2 × 150 bp). Data pre-processing and quality control were also taken into consideration prior to assembly. The pre-trimmed data quality was checked using FastQC v 0.12.1 [17]. The data were checked for base call quality distribution, % bases above Q20, Q30, %GC, and sequencing adapter contamination. The raw sequence reads were processed to remove the adapter sequences and low-quality bases using the Fastp tool (v 0.23.4) [18]. The post-trimmed QC-passed reads were assembled de novo with the Spades tool (v 3.15.5) [19]. The quality of the assembly was assessed with QUAST (v 5.2.0) [20]. The genome completeness was checked using BUSCO (v 5.8.0) [21], while genome contamination was estimated using CheckM2 (v 1.0.1) [22]. The whole-genome project containing the SRA data and assembled genome of the CBZ7 strain was deposited at GenBank under the Biosample accession SAMN62751802 (Bioproject ID PRJNA1519962).

### 2.7. Genome Annotation and Analysis

Genome annotation and functional analysis of the bacterial strain were performed using a combination of bioinformatics platforms to ensure comprehensive characterization of its predicted metabolic potential. The assembled genome was first uploaded to the Bacterial and Viral Bioinformatics Resource Center (BV-BRC): (https://www.bv-brc.org/ (accessed on 4 June 2026), where genome annotation was performed using the RASTtk pipeline, providing gene prediction, functional assignments, and protein-family classification. The obtained gene sequences were subjected to validation and functional analysis using InterProScan: https://www.ebi.ac.uk/interpro/ (accessed on 4 June 2026).

A circular genome map was generated using the BV-BRC platform following genome annotation with the RASTtk pipeline.

This initial annotation was complemented with EggNOG-mapper v2 which enabled orthology-based functional classification and assignment of Clusters of Orthologous Groups (COG), thereby providing insights into genes associated with secondary metabolism, xenobiotic degradation, and environmental adaptation [23].

GhostKOALA v2.2 was used to obtain a global KEGG-based functional classification of the predicted protein dataset and to summarize the distribution of major metabolic and cellular functional categories. KofamKOALA: https://www.genome.jp/tools/kofamkoala/ (accessed on 4 June 2026) was subsequently used for KO assignment based on profile hidden Markov models and KO-specific score thresholds, enabling the identification of candidate genes potentially associated with aromatic compound transport and oxidation, glutathione-linked detoxification, efflux systems, and transcriptional regulation. To identify metabolic pathways, the annotated coding sequences were submitted to the KEGG KOALA (KEGG Orthology And Links Annotation) tool, which assigned KEGG orthologs (KOs) to individual genes [24]. KEGG Mapper: https://www.genome.jp/kegg/mapper/ (accessed on 4 June 2026) was subsequently employed to visualize and integrate these orthologs into complete metabolic pathways, with particular attention to those involved in xenobiotic degradation, antibiotic resistance, and environmental stress responses.

Secondary metabolite biosynthetic gene clusters (BGCs) were identified using antiSMASH v 8.0.4. The analysis was conducted under relaxed strictness parameters to maximize detection sensitivity. AntiSMASH predicted several clusters, including nonribosomal peptide synthetases (NRPS), polyketide synthases (PKS), ribosomally synthesized and post-translationally modified peptides (RiPPs), arylpolyenes, and terpene precursors. Each cluster was annotated with its genomic coordinates, predicted type, and similarity to known clusters in the MIBiG database. The presence of hybrid NRPS-PKS clusters was of particular interest, as these are often associated with the production of complex bioactive metabolites and may contribute to the degradation or transformation of xenobiotics [25].

Functional annotation data generated by GhostKOALA v2.2 and KEGG Mapper were processed and visualized in R using the packages ggplot2, dplyr, and readr. The resulting plots were used to summarize the distribution of major functional categories and selected pathways associated with environmental adaptation and xenobiotic metabolism.

### 2.8. Statistical Analysis

All the experiments were performed in triplicate for each bacterial isolate and CBZ concentration. Relative growth values were calculated as the ratio between the viable bacterial count in the CBZ treatment and that in the corresponding CBZ-free control, multiplied by 100. For statistical analyses, this ratio was log_10_-transformed. To compare isolates within each CBZ concentration, separate one-way ANOVAs followed by Tukey’s honestly significant difference (HSD) tests were performed. Statistical significance was established at *p* < 0.05. Different lowercase letters indicate significant differences among bacterial isolates within the same CBZ concentration, whereas bars sharing at least one letter are not significantly different. Statistical analyses were performed using R version 4.6.1.

## 3. Results

### 3.1. Maximum Tolerance Concentration of Resistant Bacteria

Figure 1 presents the tolerance profiles of bacterial isolates evaluated in response to increasing concentrations of carbamazepine ranging from 1 to 50 mg/L.

The isolates exhibited distinct relative growth profiles across the tested CBZ concentrations (1 to 50 mg L^−1^; Figure 1). The CBZ1 strain showed values generally remaining below approximately 25%. In contrast, CBZ3 exhibited high relative growth at the lower concentrations, with values close to 100% between 1 and 2.5 mg L^−1^. CBZ3 showed significantly higher relative growth than the other isolates at 1 and 2 mg L^−1^ (*p* < 0.05); however, its relative growth progressively decreased at higher concentrations, reaching approximately 50–55% at 40 to 50 mg L^−1^.

On the other hand, CBZ6 displayed a variable concentration-dependent response with high relative growth at intermediate CBZ concentrations and a partial recovery at the highest concentration tested (45 and 50 mg L^−1^).

CBZ7 showed low relative growth at the lowest CBZ concentrations but exhibited a marked increase from 4 mg L^−1^ onward. At 30–50 mg L^−1^, CBZ7 showed significantly higher relative growth than the other isolates, as indicated by the distinct Tukey grouping letters (*p* < 0.05). This sustained response at the highest concentrations tested provided the experimental rationale for selecting CBZ7 for whole-genome sequencing. Whole-genome sequencing was therefore performed to investigate genetic features potentially associated with tolerance and adaptation to elevated CBZ concentrations.

The relative growth profiles did not follow a strictly decreasing pattern as CBZ concentration increased, indicating isolate-specific and concentration-dependent responses rather than a simple inverse relationship between CBZ concentration and bacterial growth.

### 3.2. Isolation and Screening of Indigenous Carbamazepine-Tolerant Bacteria

In this study, four bacterial strains (CBZ1, CBZ3, CBZ6, and CBZ7) were isolated and selected based on their tolerance to carbamazepine. Morphological characterization (Figure 2) revealed distinct features among the isolates. Strain CBZ1 formed small, whitish, viscous, and mucoid colonies and was identified microscopically as Gram-negative. Strain CBZ3 was characterized as Gram-variable (its stain changed between Gram-negative and positive), with rod-shaped morphology and flat colonies. Strain CBZ6 displayed smooth yellow, circular colonies with rounded edges; microscopically, it was composed of straight, rod-shaped cells with rounded ends, exhibiting a coccobacillary form and Gram-negative stain. Finally, the CBZ7 strain showed beige/grayish colonies and Gram-negative staining with a bacillus shape [6].

The biochemical identification of the strains revealed distinct metabolic profiles (Table S1), supporting their preliminary identification. Strain CBZ1 corresponds to *Enterobacter cloacae* with 96.98% identity, strain CBZ3 to *Elizabethkingia* sp. with 89.23%, strain CBZ6 to *Aeromonas hydrophila* with 99.65%, and strain CBZ7 to *Klebsiella oxytoca* with 99.22%. Considering the limited discriminatory power of phenotypic and biochemical characteristics for definitive taxonomic identification, particularly among environmental isolates, a complementary molecular identification was carried out to confirm the results obtained through phenotypic analysis [26].

All strains were found to be oxidase-negative and exhibited a diverse metabolic profile; strain CBZ7, which demonstrated the greatest tolerance, yielded positive results in most biochemical tests, suggesting the potential presence of adaptive physiological mechanisms that may confer tolerance to chemical compounds structurally analogous to those utilized in the biochemical assays.

### 3.3. Molecular Identification and Phylogenetic Analysis Based on 16S rRNA Gene Sequencing

To verify that the DNA extraction process was correct, the DNA extracted was amplified and observed in an electrophoresis gel with amplicons of approximately 1500 pb for the bacterial isolates (Figure S1), which were subsequently purified and sequenced for molecular identification.

At the time of molecular analysis, it was determined that strain CBZ1 corresponds to *Enterobacter hormaechei* with 98.88% identity, strain CBZ3 to *Paenibacillus lactis* with 99.40%, strain CBZ6 to *Aeromonas hydrophila* with 96.57%, and strain CBZ7 to *Raoultella ornithinolytica* with 98.92% (Table S1).

The discrepancy in the identification of strain CBZ3 between biochemical and molecular identification indicates that its molecular assignment should be considered tentative. Strain CBZ7 was assigned to *Raoultella ornithinolytica* according to the taxonomy and database nomenclature available at that time. Subsequent taxonomic updates currently recognize this species as *Klebsiella ornithinolytica*, with *Raoultella ornithinolytica* retained as a homotypic synonym in the NCBI Taxonomy database. Misidentification is common because *Klebsiella oxytoca* and *Raoultella ornithinolytica* belong to the same family, Enterobacteriaceae, share phenotypic traits, and exhibit similar biochemical profiles, and in some identification systems, *Raoultella* appears grouped within the genus *Klebsiella* [27]. Therefore, the whole-genome analysis of CBZ7 provided a higher-resolution taxonomic assignment than the initial biochemical and 16S rRNA-based identification.

Phylogenetic analysis based on 16S rRNA gene sequences revealed distinct taxonomic affiliations among the four environmental isolates CBZ1, CBZ3, CBZ6, and CBZ7, each clustering with reference strains from different genera. The tree, supported by 1000 bootstrap replicates, showed robust clustering patterns with values ranging from 87% to 100% (Figure 3).

CBZ7 grouped tightly with the *Raoultella ornithinolytica* reference strain, with a bootstrap value of 100%, confirming a close phylogenetic relationship. CBZ3 clustered with *Paenibacillus lactis,* also with 100% support, indicating a clear placement outside the Enterobacteriaceae. CBZ6 was positioned near *Aeromonas hydrophila*, supported by a bootstrap value of 100%, suggesting affiliation with the genus Aeromonas. CBZ1 is grouped with Enterobacter, close to *Enterobacter hormaechei*, with strong bootstrap support (99%).

### 3.4. Genome Assembly

The assembled genome of CBZ7 strain consisted of 181 contigs, with a total length of 5,671,759 bp, an average G+C content of 55.65%, consistent with values reported for related environmental bacteria [28]. The assembly had an N50 of 79,780 bp and an L50 of 16; the largest contig was 397,621 bp. These metrics describe a draft genome suitable for functional annotation.

A brief account of the different genomic features of the genome is given in Table 1.

Assessment of genome completeness using BUSCO and the Enterobacterales-specific dataset (enterobacterales_odb10; n = 440) recovered 98.2% complete BUSCOs, including 97.7% as single-copy and 0.5% as duplicated markers. Only 0.7% of the expected BUSCOs were fragmented and 1.1% were missing. CheckM2 estimated 100.0% genome completeness and 0.87% contamination for the CBZ7 assembly, supporting the high recovery of genomic content and low level of contaminating sequence.

The annotation performed with BV-BRC included 5673 protein-coding sequences (CDSs), 83 tRNA genes and 6 rRNA genes. Of the predicted proteins, 4778 received functional assignments and 885 were classified as hypothetical proteins. In addition, 1476 proteins received EC-number assignments, 1234 GO assignments and 1072 pathway assignments. In addition, 5412 and 5446 proteins were assigned to genus-specific PLFams and cross-genus PGFams, respectively. BV-BRC assigned the submitted genome to *Raoultella ornithinolytica* within the *Klebsiella/Raoultella* group.

The circular genome map provides an overview of the structural organization of the CBZ7 draft genome (Figure 4).

The distribution of CDS across the forward and reverse strands was visualized together with RNA genes and CDS showing homology to antimicrobial resistance- and virulence-associated genes. The inner tracks represent local variation in G+C content and G+C skew across the assembled contigs. As expected for a draft genome composed of 181 contigs, the map reflects the fragmented nature of the assembly while providing an integrated representation of the genomic features identified by BV-BRC/RASTtk. This genome-wide annotation provided the basis for subsequent analyses focused on functional categories, xenobiotic-associated functions, transport systems, stress response mechanisms, and metabolic pathways potentially relevant to the CBZ-tolerant phenotype.

### 3.5. Functional Annotation

Functional annotation showed a metabolically versatile genome, with genes assigned to a broad range of functions (Table S3). Candidate genes and functional groups potentially associated with xenobiotic transformation and chemical stress adaptation are summarized in Table 2.

GhostKOALA annotated 3533 of 5327 queried proteins (66.3%). The largest categories were protein families related to signaling and cellular processes (455), environmental information processing (449), proteins related to genetic information processing (448) and carbohydrate metabolism (392). Additional categories included amino acid metabolism, energy metabolism, nucleotide metabolism, cofactor and vitamin metabolism, and 33 entries assigned to “xenobiotics biodegradation and metabolism” (Figure 5).

EggNOG-mapper confirmed functional categories, with enrichment in genes related to secondary metabolism, xenobiotic degradation, and stress response, including hydrolases, oxygenases and glutathiones-related functions. Approximately 5273 protein entries were identified, including 3715 KEGG Orthology (KO) assignments, 1660 Enzyme Commission (EC) number assignments, 2676 Gene Ontology (GO) annotations, 4966 Pfam domain associated entries and 74 CAZy annotations.

### 3.6. KEGG Reconstruction and Environmental Response Functions

KEGG orthology assignments revealed mappings to several pathways potentially relevant to environmental adaptation and xenobiotic handling, including microbial metabolism in diverse environments (277 mapped objects), ABC transporters (210), two-component systems (133), degradation of aromatic compounds (32), benzoate degradation (27), glutathione metabolism (21), xylene degradation (9), fluorobenzoate degradation (7), aminobenzoate degradation (6), toluene degradation (4), nitrotoluene degradation (3) and naphthalene degradation (3) (Figure 6).

KofamKOALA and KEGG-oriented screening identified several candidate functions related to aromatic xenobiotic metabolism, transport, detoxification and stress response. Notable candidates included a 4-hydroxybenzoate transporter (K08195), p-hydroxybenzoate 3-monooxygenase/PobA (K00481), an AraC-family transcriptional activator of pobA (K18954), and components of the AaeXAB aromatic-acid efflux system, including AaeA (K15548) and AaeB (K03468). Additional assignments included quercetin 2,3-dioxygenase (K06911), an extradiol-type 4,5-DOPA dioxygenase (K15777), glutathione S-transferases (K00799), glutathione reductase (K00383), gamma-glutamyltranspeptidase/glutathione hydrolase (K00681), S-formylglutathione hydrolase (K01070), and glutathione-regulated Kef potassium-efflux proteins (K11745/K11747). Mar/Sox/Rob- (K05804), TetR/AcrR- (K05501), and LysR-family regulators (K18900/K03566) were also represented.

### 3.7. Secondary Metabolite Biosynthetic Gene Clusters

antiSMASH analysis detected six biosynthetic gene clusters (BGCs) in the CBZ7 draft genome, including an arylpolyene region, an NRP-metallophore/NRPS-like/NRPS region with high similarity to the frederiksenibactin biosynthetic cluster, a terpene-precursor region, a RiPP-like region, an azole-containing RiPP region and a hybrid NRPS/type I polyketide synthase region (Table 3). The arylpolyene and frederiksenibactin-like regions are the most readily interpretable because antiSMASH returned high similarity to characterized clusters. These biosynthetic gene clusters should be discussed in terms of ecological fitness, stress tolerance, or microbial competitiveness. 

## 4. Discussion

### 4.1. Maximum Tolerance Concentration of Resistant Bacteria

Carbamazepine is widely regarded as a persistent pharmaceutical contaminant because of its chemical stability and relatively low susceptibility to conventional biological treatment. Recent assessments continue to identify CBZ among the pharmaceuticals that are frequently detected in aquatic and groundwater environments, emphasizing its environmental persistence and limited removal during conventional wastewater treatment [29].

Bacteria inhabiting contaminated environments may undergo selection for physiological traits that allow persistence under chemical stress, including changes in membrane transport, efflux capacity, oxidative-stress responses, and xenobiotic detoxification mechanisms. More recent studies have therefore combined enrichment and isolation approaches to distinguish growth under pharmaceutical exposure. In this context, Pápai et al. selectively enriched groundwater biofilm communities for microorganisms capable of interacting with diclofenac, ibuprofen, and carbamazepine and subsequently isolated bacterial strains associated with pharmaceutical removal. Their work illustrates that the capacity to transform pharmaceuticals can be restricted to particular taxa or strains even within microbial communities previously exposed to these contaminants [30]. This strain-specific behavior is consistent with the markedly different tolerance patterns observed among CBZ1, CBZ3, CBZ6, and CBZ7 in the present study, across the range of carbamazepine concentrations tested.

Although a progressive decrease in bacterial growth may be expected as pollutant concentration increases, microbial responses to chemical stress are not necessarily monotonic. Sublethal exposure can induce strain-specific stress responses, changes in membrane transport, cellular repair mechanisms, and metabolic adjustments, whereas higher concentrations may exert stronger inhibitory effects. Non-monotonic bacterial growth responses have been reported for other antimicrobial compounds, including growth stimulation at subinhibitory concentrations followed by inhibition at higher concentrations [31,32]. In the specific case of CBZ, exposure has been shown to induce reactive oxygen species production, activate the SOS response, alter membrane permeability, and modify the expression of oxidative-stress regulators in Gram-negative bacteria [33]. These responses may differ among bacterial taxa and could contribute to the distinct concentration-dependent profiles observed for CBZ1, CBZ3, CBZ6, and CBZ7. However, the present study evaluated viable counts at a single endpoint and did not measure growth kinetics, gene expression, cellular stress markers, or CBZ depletion. Therefore, the observed fluctuations should be interpreted conservatively as strain- and concentration-specific tolerance responses rather than evidence of hormesis, physiological adaptation, or CBZ transformation.

Among the four isolates, strain CBZ7 showed the most consistently high tolerance, maintaining comparatively elevated growth throughout the concentration range, including the highest CBZ concentrations evaluated (50 mg L^−1^). CBZ6 and CBZ3 displayed intermediate responses, whereas CBZ1 showed substantially lower tolerance across most experimental conditions. These differences indicate that tolerance to CBZ is strain-dependent and may reflect distinct physiological strategies for coping with pharmaceutical-induced chemical stress; the ability of CBZ7 to maintain growth under increasing CBZ concentrations is therefore particularly relevant from an ecological perspective. Reviews of pharmaceutical removal have similarly emphasized that biological removal efficiency is highly compound- and microorganism-dependent, particularly for persistent pharmaceuticals such as CBZ [34].

Such tolerance could arise from mechanisms that reduce intracellular exposure to the compound or mitigate its cellular effects rather than from direct degradation [3]. This interpretation is consistent with the subsequent genomic characterization of CBZ7, which revealed functions related to membrane transport, efflux, oxidative-stress response, detoxification, and xenobiotic-associated metabolism.

Recent research also supports the importance of studying microbial communities and combined biological systems for recalcitrant pharmaceuticals. For example, coupled microalgal–bacterial biofilms have been investigated for the removal of CBZ and other pharmaceuticals, reflecting growing interest in microbial interactions as a strategy for improving the treatment of compounds that are poorly removed by individual organisms [35].

Growth in the presence of a contaminant should not be interpreted by itself as evidence of its degradation. Bioremediation is also constrained by the biodegradability of the target compound and by the time required for complete transformation; therefore, the selection of microorganisms with suitable physiological and metabolic characteristics is a critical step when evaluating their potential environmental application [2].

Overall, the tolerance assay allowed discrimination among isolates with markedly different responses to CBZ exposure and supported the selection of CBZ7 for further characterization. Its ability to sustain high relative tolerance across the tested concentration range indicates a robust phenotype under CBZ stress.

These findings suggest that isolates displaying strong tolerance, such as CBZ7, may be valuable for subsequent studies investigating either individual biotransformation mechanisms or their contribution within controlled microbial consortia to determine whether this tolerance phenotype is accompanied by actual biotransformation of the pharmaceutical.

### 4.2. Isolation and Screening of Indigenous Carbamazepine-Tolerant Bacteria

Culture media provide microorganisms with nutrients required for growth, while under selective conditions some microorganisms may additionally exploit organic pollutants through specific metabolic mechanisms [2]. Biochemical testing further differentiated the isolates through reactions associated with carbohydrate utilization, amino acid metabolism, hydrolytic activity, and other metabolic traits. These tests are useful for preliminary bacterial discrimination because combinations of reactions, rather than individual biochemical traits, provide characteristic phenotypic profiles. Nevertheless, environmental isolates may exhibit atypical reactions, and phenotypic identification systems should therefore be regarded as presumptive when species-level discrimination is required.

Among the isolates, CBZ1 exhibited a relatively broad metabolic profile, and strain CBZ1 exhibited the closest similarity to *Enterobacter cloacae* (96.98%). According to Bergey’s Manual, members of *Enterobacter* are Gram-negative, facultatively anaerobic rods that ferment glucose, and most strains are positive for the Voges–Proskauer, arginine dihydrolase, and ornithine decarboxylase tests, while generally being negative for indole, lysine decarboxylase, and H_2_S production [36]. These characteristics show substantial agreement with the profile obtained for CBZ1, particularly the positive ORN, VP, and ARG reactions and the negative LYS, IND, and H_2_S tests. The environmental origin of CBZ1 is also compatible with the ecology of the genus, since *Enterobacter* species are widely distributed in terrestrial and aquatic environments as well as in association with plants, animals, and clinical settings [36].

CBZ3 exhibited the most restricted biochemical profile of the four isolates. Positive reactions were limited primarily to ONPG, gelatin hydrolysis, and arabinose utilization, whereas the remaining biochemical reactions were negative. Recent literature emphasizes that phenotypic identification of *Elizabethkingia* is problematic because closely related species can be misidentified by conventional biochemical systems and database-dependent identification platforms [37]. Therefore, the comparatively low biochemical identification score obtained for CBZ3 is consistent with the limited discriminatory power of the phenotypic profile observed in this isolate.

Strain CBZ6 showed the highest identity among the isolates evaluated, displaying 99.65% similarity to *Aeromonas hydrophila* and a markedly different biochemical pattern, with positive reactions for lysine decarboxylase, H_2_S production, glucose, mannitol, xylose, ONPG, indole, Voges–Proskauer, citrate utilization, tryptophan deaminase, gelatin hydrolysis, sucrose, arabinose, salicin, and arginine. Members of the genus Aeromonas are ubiquitous in aquatic environments and have been recovered from bottled water, chlorinated water, groundwater and highly polluted aquatic systems. This broad environmental distribution is consistent with the aquatic origin of the isolates obtained in the present study [38].

CBZ7 showed the broadest positive biochemical profile among the isolates, because only ornithine decarboxylase, H_2_S production, Voges–Proskauer, tryptophan deaminase, and arginine were negative. The biochemical identification system assigned this profile most closely to *Klebsiella oxytoca*. Bergey’s Manual describes *Klebsiella* as Gram-negative, predominantly non-motile, capsulated rods that form smooth, circular, often glistening colonies and exhibit both respiratory and fermentative metabolism [39].

The predominance of Gram-negative bacteria among the CBZ-tolerant isolates may also be relevant from the perspective of environmental stress tolerance. Gram-negative bacteria possess an outer membrane which can provide an additional permeability barrier against several toxic compounds. This structural organization has been associated with increased tolerance to some antimicrobial and chemical stresses [2].

The biochemical assignments obtained here were considered preliminary and were subsequently complemented with molecular approaches for more robust taxonomic identification.

### 4.3. Molecular Identification and Phylogenetic Analysis Based on 16S rRNA Gene Sequencing

The molecular identification based on 16S rRNA gene sequence comparison revealed taxonomic diversity among the bacterial isolates selected under carbamazepine exposure. According to the identification criterion adopted in the present study, bacterial sequences showing ≥98.6% identity are considered highly likely to belong to the same species-level relationship, whereas lower similarities were interpreted more conservatively as supporting assignment at broader taxonomic levels [40,41]. According to these criteria and based on the taxonomy available at the time of molecular analysis, strains CBZ1, CBZ3 and CBZ7 were identified as *Enterobacter hormaechei*, *Paenibacillus lactis* and *Raoultella ornithinolytica* with gene sequencing identities of 98.88, 99.4 and 98.92%, respectively. CBZ6 was identified as *Aeromonas hydrophila* with 96.57%.

The identification of an *Enterobacter*-related isolate among the CBZ-tolerant bacteria is environmentally relevant because members of this genus have demonstrated the capacity to transform structurally diverse xenobiotics, including pharmaceutical contaminants. Enterobacter *hormaechei* D15, isolated from activated sludge, was reported to transform the non-steroidal anti-inflammatory drug diclofenac, reaching an elimination rate of 52.8%, which increased to approximately 82% in the presence of glucose as an additional carbon source [42]. A subsequent study using a bacterial consortium containing *Enterobacter hormaechei* D15 and an *Enterobacter cloacae* isolate reported substantial diclofenac removal [42,43], providing relevant evidence that members of the genus *Enterobacter* can tolerate and transform structurally diverse pharmaceutical contaminants.

More recent evidence further supports the potential involvement of *Enterobacter* in pharmaceutical biodegradation. An *Enterobacter* sp. isolate was reported to remove approximately 85.9% of acetaminophen at an initial concentration of 300 mg L^−1^ within eight days, with transformation intermediates identified by analytical approaches [44]. Additionally, *Enterobacter* sp. APAP_BS8 achieved approximately 88% acetaminophen degradation in microcosms, while integrated genomic, proteomic, and metabolomic analyses identified oxidoreductases and other enzyme systems potentially associated with the transformation pathway [44].

The identification of CBZ3 as *Paenibacillus lactis* is environmentally relevant because members of the genus *Paenibacillus* are widely distributed in soil, water, plant-associated environments, and other ecological niches, where they display substantial physiological and metabolic diversity. Although information specifically linking *P. lactis* to pharmaceutical transformation remains limited, members of the genus have been associated with the degradation or transformation of structurally diverse organic compounds and with adaptation to chemically stressed environments. This metabolic versatility is also reflected in recent genomic studies. For example, a *Paenibacillus caseinilyticus* strain isolated from soil was recently characterized as a potential acetaminophen-removing bacterium; genome annotation revealed several amidase genes potentially involved in hydrolysis of the amide bond of acetaminophen [45]. This finding provides recent evidence that members of *Paenibacillus* may harbor enzymatic functions relevant to the transformation of pharmaceutical contaminants.

On the other hand, the identification of CBZ6 as *Aeromonas hydrophila* is consistent with the aquatic origin of isolates. Species of the genus *Aeromonas* are common inhabitants of freshwater, wastewater, and other natural and engineered aquatic environments, where they can persist under diverse physicochemical conditions. A recent systematic review identified *Aeromonas* among the environmentally relevant bacterial genera frequently recovered from wastewater and surface waters, emphasizing their capacity to survive and proliferate in aquatic systems subjected to anthropogenic pressures [46].

The ecological versatility of *A. hydrophila* also includes the capacity to respond to a broad range of chemical stresses. For example, an environmental multidrug-resistant *A. hydrophila* recovered from wastewater showed reduced susceptibility to the quaternary ammonium compound benzalkonium chloride. Transcriptomic and physiological analyses indicated that this phenotype involved multiple cellular responses rather than a single resistance determinant, including changes associated with membrane functions, transport, and stress [47]. Although benzalkonium chloride differs structurally and functionally from carbamazepine, this study illustrates the capacity of environmental *A. hydrophila* strains to tolerate anthropogenic chemical stress through complex physiological responses.

The identification of CBZ7 as *Raoultella ornithinolytica* is also environmentally plausible, as this species has been recovered from contaminated aquatic and terrestrial environments and has demonstrated tolerance to diverse environmental pollutants. For example, a strain isolated from metal-contaminated river water showed substantial tolerance and removal capacity for several heavy metals, supported by genes associated with metal resistance and transport [48]. In addition, genome studies have demonstrated considerable metabolic potential in *R. ornithinolytica* for the transformation of aromatic environmental contaminants, including phthalate esters [49]. These observations provide an environmental context for the high CBZ tolerance observed in CBZ7; however, its specific genomic features potentially associated with xenobiotic tolerance and transformation are discussed in greater detail below.

Taken together, the molecular results indicate that CBZ tolerance was observed among phylogenetically distinct Gram-negative bacterial taxa rather than being restricted to a single lineage. This diversity suggests that adaptation to CBZ exposure may arise through different physiological strategies, potentially involving membrane transport, stress response systems, redox metabolism or co-metabolic transformation. However, the present 16S rRNA data establish taxonomic relatedness rather than degradation capacity, and direct CBZ biotransformation should therefore be evaluated independently through chemical quantification and metabolite analysis.

Among the isolates, CBZ7 was selected for whole-genome characterization for a higher-resolution taxonomic framework and to explore functional traits potentially associated with xenobiotic tolerance and aromatic compound metabolism.

### 4.4. Genome Assembly

The experimental design selected microorganisms that remained viable and grew during exposure to carbamazepine, after which one of the most tolerant isolates, CBZ7, was characterized genomically. This type of enrichment is biologically meaningful because pharmaceutical exposure can select organisms with stress response and transformation capabilities.

In this context, the assembly of CBZ7 is considered a draft genome, with acceptable N50 and L50 values that indicate assembly contiguity. However, these metrics describe fragmentation rather than overall genome accuracy or completeness and should not be interpreted independently of other assembly characteristics [50].

In the case of CBZ7, the assembly comprised 181 contigs with an N50 of 79,780 bp and an L50 of 16, while BUSCO analysis using the lineage-specific enterobacterales_odb10 dataset recovered 98.2% of the 440 expected conserved orthologs. Of these, 97.7% were present as complete single-copy genes and only 0.5% were duplicated, whereas 0.7% were fragmented and 1.1% were missing. The high proportion of complete single-copy BUSCOs, together with the small proportion of fragmented and missing markers, indicates that most of the conserved genomic content expected for members of Enterobacterales was recovered despite the fragmentation of the assembly. Complementarily, CheckM2 estimated 100% genome completeness and only 0.87% contamination. The concordance between these independent approaches indicates that the assembly recovered nearly all the expected genomic content with minimal evidence of contamination.

The overall genome size and nucleotide composition further support the consistency of the assembly. The CBZ7 genome comprised 5,671,759 bp with a G+C content of 55.65%. These values fall within the range reported for *Raoultella ornithinolytica*. Villacis et al. reported genomes of approximately 5.58–5.93 Mb with G+C contents of 55.3–55.7% [51], whereas Alam reported a 5.4 Mb *R. ornithinolytica* draft genome with a G+C content of 55.9% [52]. Thus, the genome size and base composition of CBZ7 are consistent with previously sequenced members of this taxonomic group.

Additionally, Alam obtained 145 contigs with an N50 of 89,898 bp [52], while Villacis et reported assemblies containing 39–97 contigs with N50 values ranging from approximately 196 to 739 kb [51]. Nevertheless, differences in sequencing platforms, read depth, library characteristics, assembly algorithms, and repetitive genomic regions can substantially affect contiguity metrics, making direct comparisons among assemblies dependent on the methodological context.

### 4.5. Integrated Interpretation of Genomic Findings

The taxonomic context of CBZ7 is compatible with environmental metabolic versatility, although it does not independently demonstrate CBZ degradation. Feng et al. used genome mining and gene activation to demonstrate complete phthalate ester biodegradation in *Raoultella ornithinolytica* XF201 [49]. Jiang reported extensive chloramphenicol biotransformation by *R. ornithinolytica* CT3, identifying 22 transformation products and integrating chemical analyses with genomic predictions for acetyltransferase, hydrolase, nitroreductase, dehalogenase and protocatechuate-dioxygenase functions [53]. These studies support broad stress response within this lineage, while remaining contextual rather than direct evidence for CBZ transformation by CBZ7.

The CBZ7 genome contains several functionally coherent systems that can plausibly contribute to survival under xenobiotic stress: aromatic compound transport and efflux, oxygenases and dioxygenases, glutathione-dependent detoxification, redox-maintenance enzymes and global transcriptional regulators. The PobA-associated functions and *AaeXAB* efflux components are particularly relevant because they indicate that CBZ7 encodes complementary functions associated with the uptake, oxidative metabolism, and extrusion of aromatic-acid substrates. In bacterial aromatic-acid metabolism, 4-hydroxybenzoate transport can be coupled to its hydroxylation by *PobA* to form protocatechuate, whereas the *AaeXAB* system has been experimentally associated with the efflux of hydroxylated aromatic carboxylic acids, including 4-hydroxybenzoate [54,55].

A useful next step would be to compare transcription of *pobA*, *aaeA/aaeB*, selected glutathione S-transferases, oxidative-stress genes and global regulators between CBZ-exposed and unexposed cells. Such targeted RT-qPCR or transcriptomic data would connect the genomic repertoire to the experimentally observed tolerance phenotype and help prioritize enzymes for subsequent biochemical assays.

Although the genus Raoultella has traditionally been studied mainly in clinical contexts because of the opportunistic pathogenic potential and antimicrobial resistance reported for some members of the Raoultella group [27], the genomic analysis of strain CBZ7 revealed a broad repertoire of functions potentially relevant to xenobiotic handling and environmental stress adaptation [49]. These included monooxygenases, oxygenases/dioxygenases, oxidoreductases, hydrolases, transferases, transport systems, and glutathione-associated detoxification mechanisms, together with functions related to aromatic compound metabolism [53]. Such a functional profile is compatible with a potential capacity for xenobiotic transformation, including carbamazepine biotransformation.

Previous studies have shown that the initial transformation of CBZ may involve oxidative, hydroxylation, epoxidation, and deamidation reactions mediated by enzymes such as biphenyl dioxygenases, amide hydrolases/deamidases, and cytochrome P450 systems. In microbial model communities, genes associated with deamidation and hydroxylation pathways have been linked to enhanced CBZ removal, while metagenomic analyses of CBZ-degrading systems have also shown enrichment of functions involved in aromatic compound metabolism [56,57].

Wang et al proposed that the initial deamidation of CBZ forms iminostilbene, followed by dioxygenase-mediated oxidation, subsequent ring-opening and condensation reactions involving lyase- and aldolase-associated steps, ultimately leading to the formation of diphenylamine [1]. Accordingly, the presence of oxidases, dioxygenases, hydrolases or deamidase-related functions has been considered potentially relevant when evaluating bacterial candidates for CBZ transformation. Earlier experimental work also demonstrated that biphenyl dioxygenase from *Paraburkholderia xenovorans* LB400 can transform CBZ into dihydroxylated derivatives [58]. Experimental quantification of CBZ depletion, together with the identification of transformation products by appropriate analytical methods, would therefore be required to determine whether these genomic functions contribute directly to CBZ biotransformation.

Oxidoreductases play an important role in the detoxification and transformation of organic compounds by mediating oxidation–reduction reactions and electron transfer between substrates and acceptors [2]. However, the metabolic response to pollutants varies substantially among bacterial taxa and strains, and the presence of a contaminant-tolerant phenotype does not necessarily imply an equivalent degradation capacity. Consequently, the selection of microorganisms for bioremediation must consider both their environmental tolerance and their demonstrated metabolic performance under the conditions of interest [2].

The functional profile of CBZ7 also included glutathione-associated detoxification systems, membrane transporters, multidrug and aromatic-acid efflux functions, oxidative-stress response mechanisms, and global transcriptional regulators. Such systems are commonly involved in maintaining cellular homeostasis under chemical stress by limiting intracellular accumulation of toxic compounds, mitigating oxidative damage, and regulating adaptive responses [3,59,60].

From a biotechnological perspective, the genomic repertoire of CBZ7 identifies candidate functions of potential interest associated with tolerance to xenobiotic stress. However, members of the *Raoultella* group have been associated with opportunistic pathogenicity and antimicrobial resistance [27], which should be considered in any future application-oriented assessment. Therefore, the biotechnological relevance of CBZ7 may lie primarily in the identification and characterization of specific enzymes or metabolic functions rather than in the direct environmental use of the strain.

## 5. Conclusions

Enrichment of water from the Machángara River in CBZ-supplemented medium yielded four bacterial isolates with distinct phenotypic, biochemical, and molecular characteristics. Viable-count assays revealed strain- and concentration-specific relative growth profiles rather than a uniform decrease in growth as CBZ concentration increased. Culturable cells were detected at the highest concentration tested (50 mg L^−1^), demonstrating that the isolates tolerated CBZ under the experimental conditions. Among them, CBZ7 exhibited significantly higher relative growth at elevated CBZ concentrations (30–50 mg L^−1^) and was therefore selected for whole-genome sequencing.

Strain CBZ7 was identified as *Raoultella ornithinolytica* with a 5.67 Mb draft genome comprising 181 contigs. Complementary genomic analyses identified predicted functions associated with membrane transport, efflux, environmental sensing, oxidative metabolism, glutathione-linked detoxification, aromatic compound metabolism, and secondary metabolism. These findings provide a plausible genomic basis for adaptation to chemical stress and identify candidate functions that may contribute to the survival of CBZ7 under CBZ exposure.

However, no complete or uniquely CBZ-specific catabolic pathway was identified; therefore, the present results support CBZ tolerance. Further studies combining time-resolved growth measurements, analytical quantification of CBZ and its transformation products, and functional validation of candidate genes are required to determine the mechanisms underlying CBZ7 tolerance and assess its potential relevance for bioremediation.

## Figures and Tables

**Figure 1:**
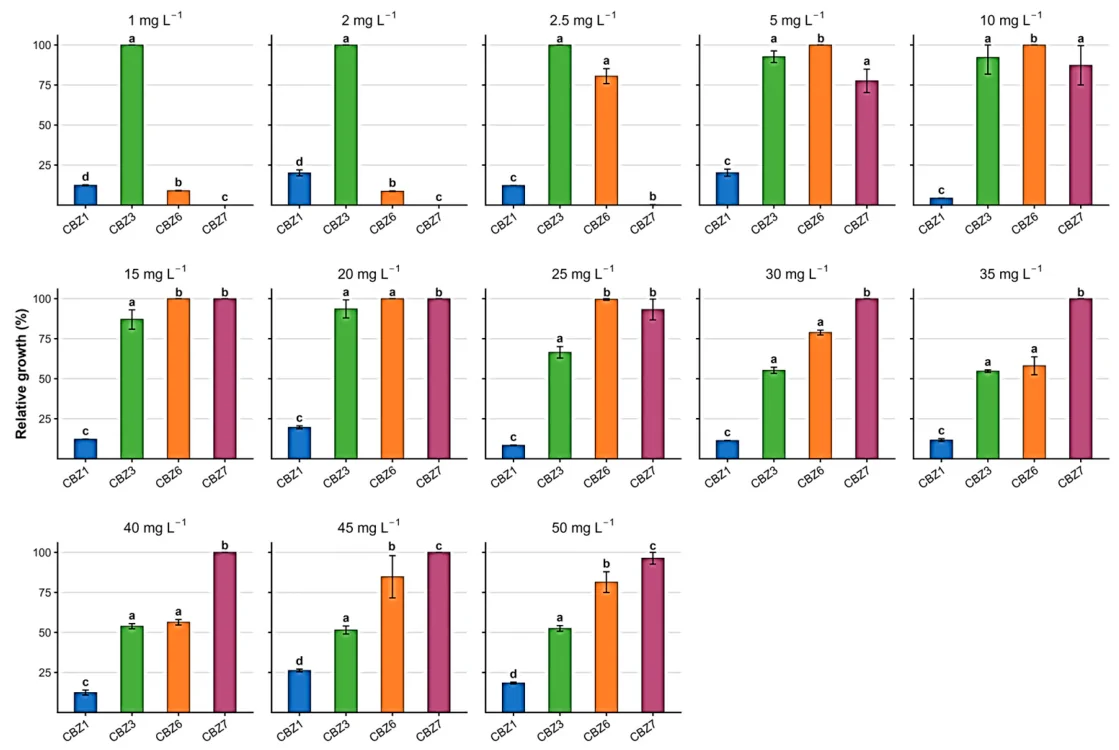
Relative growth of the four bacterial isolates after 7 days of exposure to increasing CBZ concentrations. Note: Each panel represents one CBZ concentration; the x-axis indicates the bacterial isolates, and the y-axis shows relative growth compared with the corresponding nutrient-rich CBZ-free control. Bars represent the mean ± standard deviation of three replicates (*n* = 3). Different lowercase letters indicate significant differences among bacterial isolates within the same CBZ concentration, based on one-way ANOVA followed by Tukey’s honestly significant difference (HSD) test (*p* < 0.05). Bars sharing at least one letter are not significantly different.

**Figure 2:**
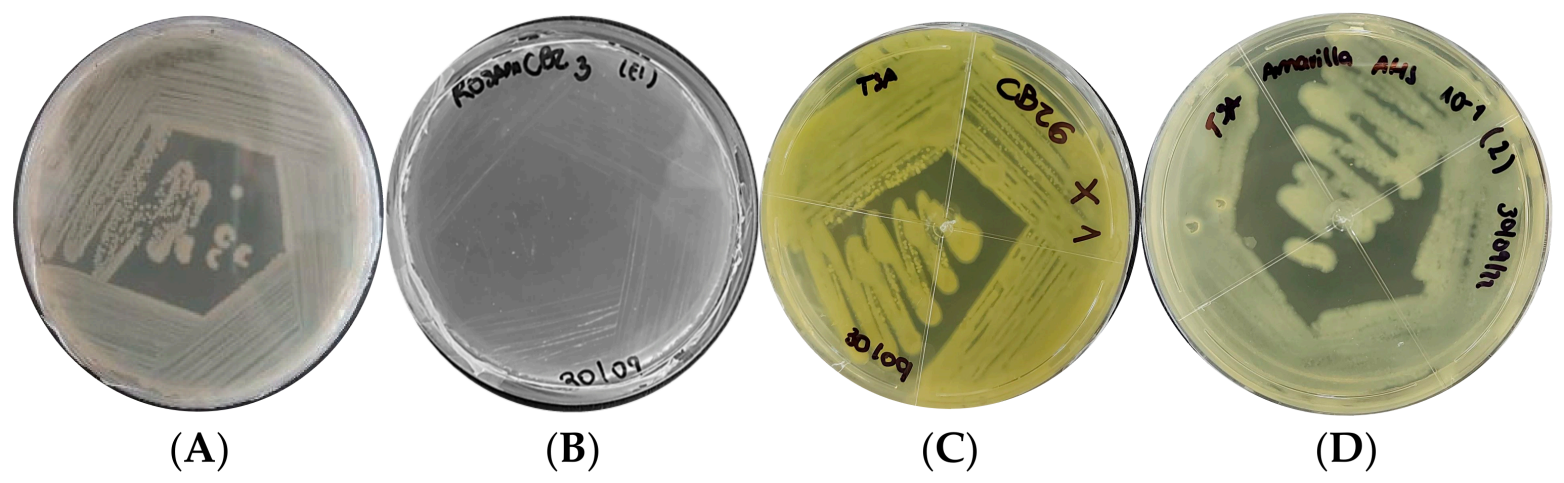
Colony morphology of carbamazepine-tolerant bacterial isolates. (**A**) CBZ1, (**B**) CBZ3, (**C**) CBZ6, and (**D**) CBZ7.

**Figure 3:**
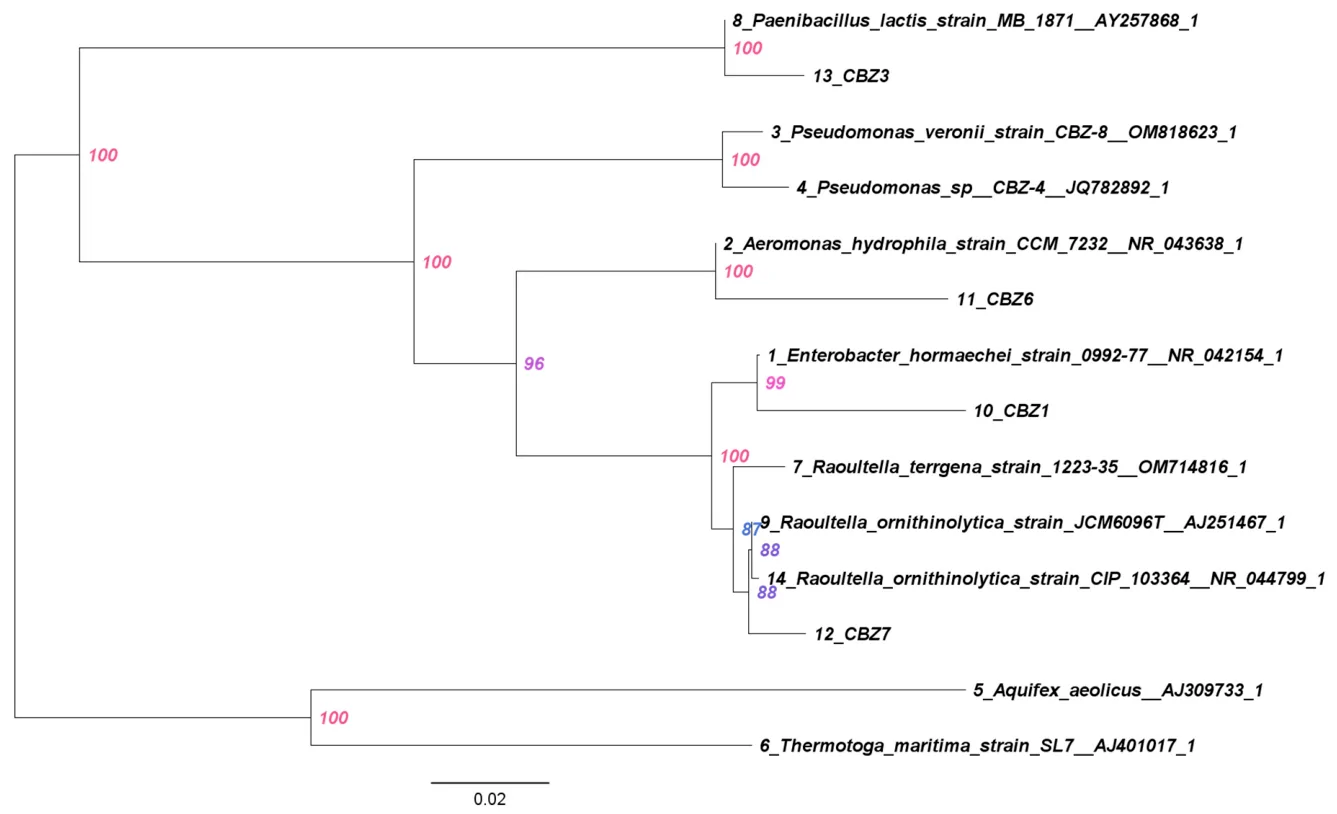
Phylogenetic tree based on 16S rRNA gene sequences showing the evolutionary relationships among environmental bacterial isolates and reference strains. Note: maximum likelihood tree indicating the phylogenetic position of the CBZ1, CBZ3, CBZ6, and CBZ7 strains. Numbers at nodes indicate bootstrap value (1000 replicates). Scale bars represent substitutions per nucleotide position. *Raoultella ornithinolytica* is retained in the figure because this was the taxonomic designation used at the time of the analysis; the species is currently recognized by NCBI as *Klebsiella ornithinolytica*.

**Figure 4:**
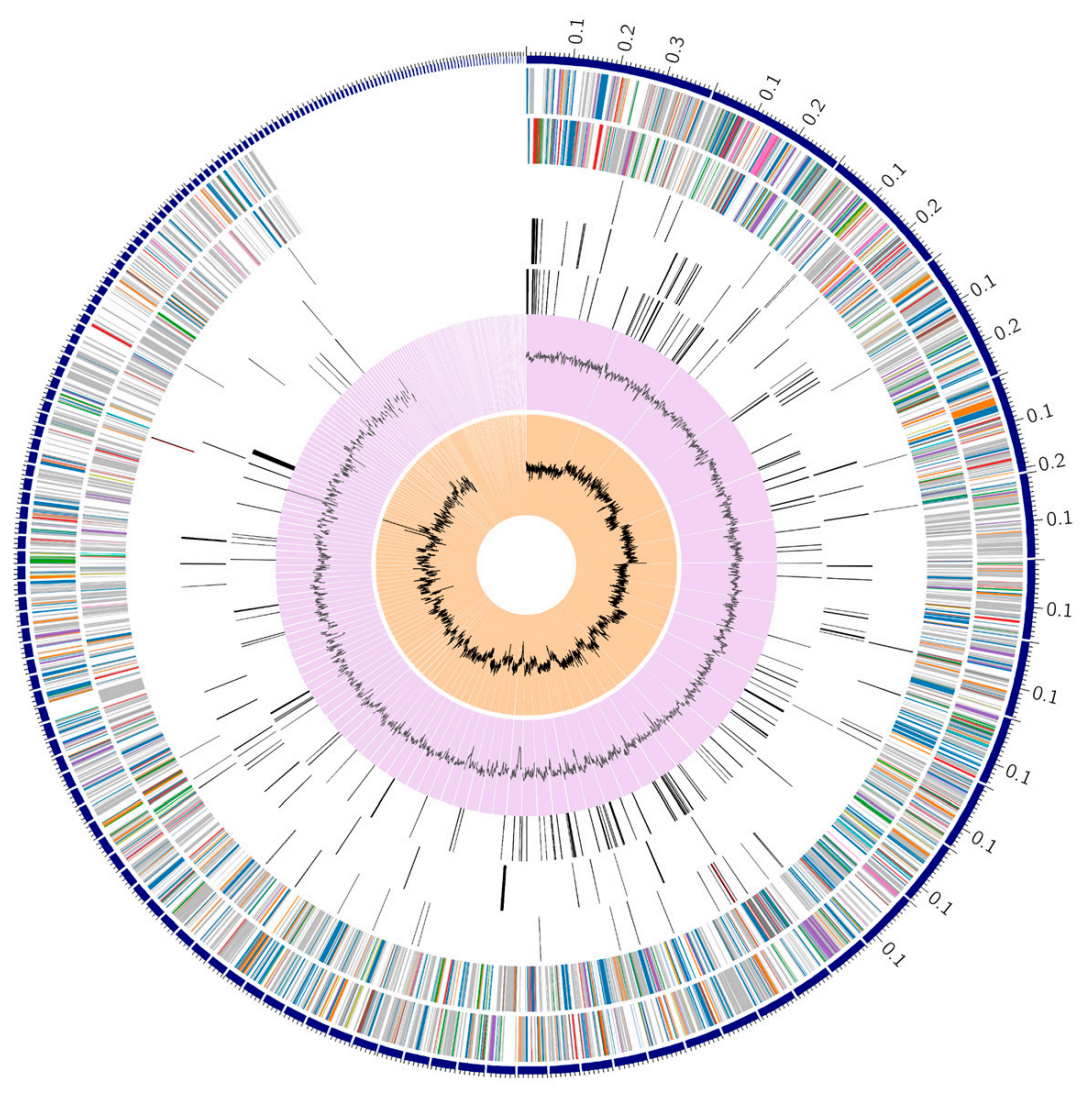
Circular representation of the draft genome of strain CBZ7 based on the BV-BRC/RASTtk annotation. Note: From outer to inner tracks, the map summarizes contigs, coding sequences (CDSs) on the forward and reverse strands, RNA genes, CDS with homology to antimicrobial resistance and virulence-associated genes, G+C content and G+C skew.

**Figure 5:**
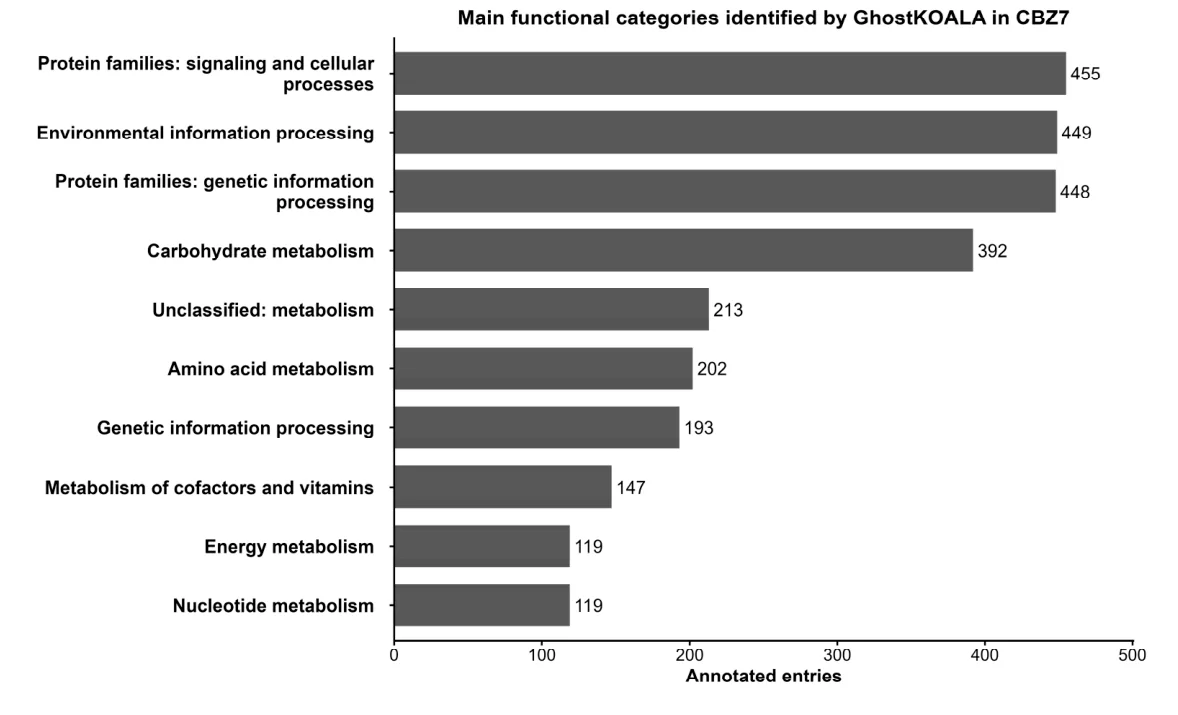
Main functional categories detected by GhostKOALA in CBZ7. Note: Values represent the number of annotated entries assigned to each GhostKOALA functional category.

**Figure 6:**
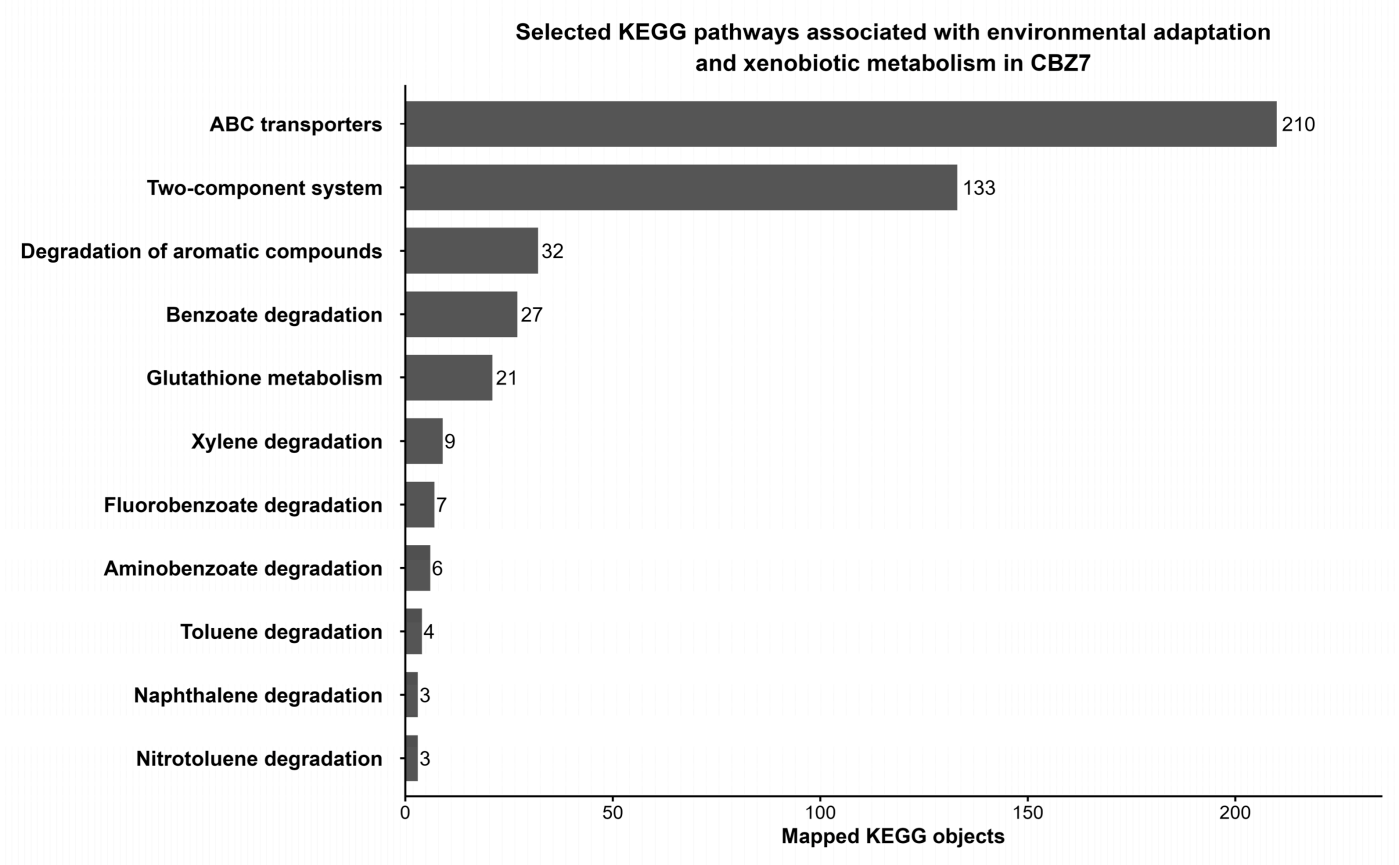
Selected KEGG pathways with potential relevance to environmental adaptation, aromatic compound metabolism, transport and detoxification in CBZ7.

**Table 1:** Summary of whole-genome assembly quality metrics and genomic annotation features of *Raoultella ornithinolytica* strain CBZ7.

Features	CBZ7
Assembly size	5,671,759 bp
Number of contigs	181
Read length	126
Contigs N50 (bp)	79,780 bp
Contigs L50 (bp)	16
Completeness	98.2%
Contamination	0.87%
GC%	55.65
CDS	5673
Gene	5394
tRNA	83
rRNA	6

**Table 2:** Functional annotation categories potentially associated with xenobiotic transformation and chemical stress response in strain CBZ7.

Gene/Function Category	Number of Matching EggNOG Annotations	Proposed/General Function Supported by Annotation
Hydrolases/esterases/amidases	201	Hydrolytic cleavage reactions; may participate in transformation of diverse organic substrates
Dioxygenases	24	Incorporation of molecular oxygen into substrates; several dioxygenases are involved in aromatic compound transformation
Monooxygenases/oxygenases/hydroxylases	51	Oxidation and hydroxylation reactions potentially relevant to xenobiotic transformation
Oxidoreductases/dehydrogenases/reductases	313	Oxidation–reduction reactions and cellular redox metabolism
Glutathione-related proteins	22	Detoxification and oxidative-stress response
Aromatic compound-related annotations	33	Functions associated with transport or metabolism of aromatic compounds
Transporters/efflux-related proteins	702	Uptake and efflux of diverse compounds; potentially relevant to chemical tolerance

Note: Counts were obtained by keyword-based screening of eggNOG-mapper annotations.

**Table 3:** Predicted biosynthetic gene clusters identified in the whole-genome assembly of Raoultella ornithinolytica strain CBZ7 and their similarity to characterized MIBiG clusters.

Cluster ID (Region)	Contig (NODE)	Type (NRPS, PKS, RiPP, etc.)	Coordinates Within Contig (bp)	Similarity to Known Clusters (MIBiG)
Region 1.1	NODE_1	Arylpolyene	153,346–196,654	Aryl polyenes—high similarity
Region 9.1	NODE_9	NRP-metallophore/NRPS-like/NRPS	87,642–142,410	Frederiksenibactin—high similarity
Region 11.1	NODE_11	Terpene-precursor	45,712–66,611	No close characterized cluster reported
Region 24.1	NODE_24	RiPP-like	1–8356	No close characterized cluster reported
Region 44.1	NODE_44	Azole-containing RiPP	1539–27,817	No close characterized cluster reported
Region 75.1	NODE_75	NRPS/type I PKS (T1PKS)	1–19,903	No close characterized cluster reported

Note: Similarity assignments were obtained from antiSMASH comparison against characterized biosynthetic gene clusters in the MIBiG database. NRPS, nonribosomal peptide synthetase; PKS, polyketide synthase; RiPP, ribosomally synthesized and post-translationally modified peptide.

## Data Availability

Partial 16S rRNA gene sequences were deposited in GenBank under the following accession numbers: CBZ1 (PV916048), CBZ3 (PV916049), CBZ6 (PV916050), and CBZ7 (PV916051). The draft genome of strain CBZ7 is available under BioSample accession SAMN62751802 and BioProject PRJNA1519962.

## Supplementary Materials

The following supporting information can be downloaded at: https://www.mdpi.com/article/10.3390/microorganisms14102305/s1. Figure S1: 1% agarose gel electrophoresis of 16S RNA gene of the samples after PCR amplification; Table S1: Biochemical reactions of strains under study; Table S2: Molecular identification of carbamazepine-tolerant bacterial isolates based on 16S rRNA gene sequencing; Table S3: Draft genome annotation of strain CBZ7.

## Abbreviations

The following abbreviations are used in this manuscript:

Abbreviation	Definition
EC	Enzyme Commission
BGC	Biosynthetic Gene Cluster
NRPS	Nonribosomal Peptide Synthetase
PKS	Polyketide Synthase
RiPP	Ribosomally Synthesized and Post-Translationally Modified Peptide
AMR	Antimicrobial Resistance
GST	Glutathione S-Transferase
HPLC	High-Performance Liquid Chromatography
LC–MS	Liquid Chromatography–Mass Spectrometry
CBZ	Carbamazepine
MSM	Mineral Salts Medium
MTC	Maximum Tolerance Concentration
CFU	Colony-Forming Units
PCR	Polymerase Chain Reaction
WGS	Whole-Genome Sequencing
CDS	Coding Sequence
BUSCO	Benchmarking Universal Single-Copy Orthologs
KEGG	Kyoto Encyclopedia of Genes and Genomes
KO	KEGG Orthology
GO	Gene Ontology
