# Supplementary material for: Isolation, Identification and Genomic Characterization of Carbamazepine-Tolerant Bacteria from Contaminated Aquatic Environments
Source: Microorganisms. 2026 Oct 10;14(10):2305. doi: 10.3390/microorganisms14102305 (PMC13707586; doi:10.3390/microorganisms14102305)
Supplement: Supplementary file 1 [file microorganisms-14-02305-s001.zip › Figure S1.pdf]

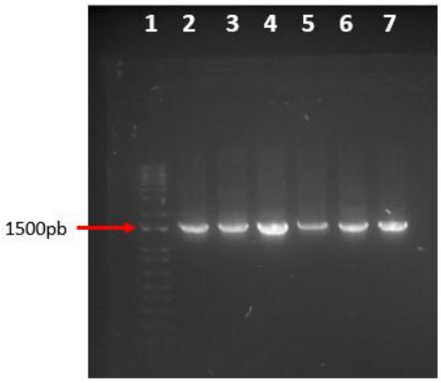

Figure S1. 1% agarose gel electrophoresis of 16s RNA gene of the samples after PCR amplification.

Note: 1: Ladder.: 2-3: CBZ3. 4: CBZ1, 5: CBZ6. 6-7: CBZ7.

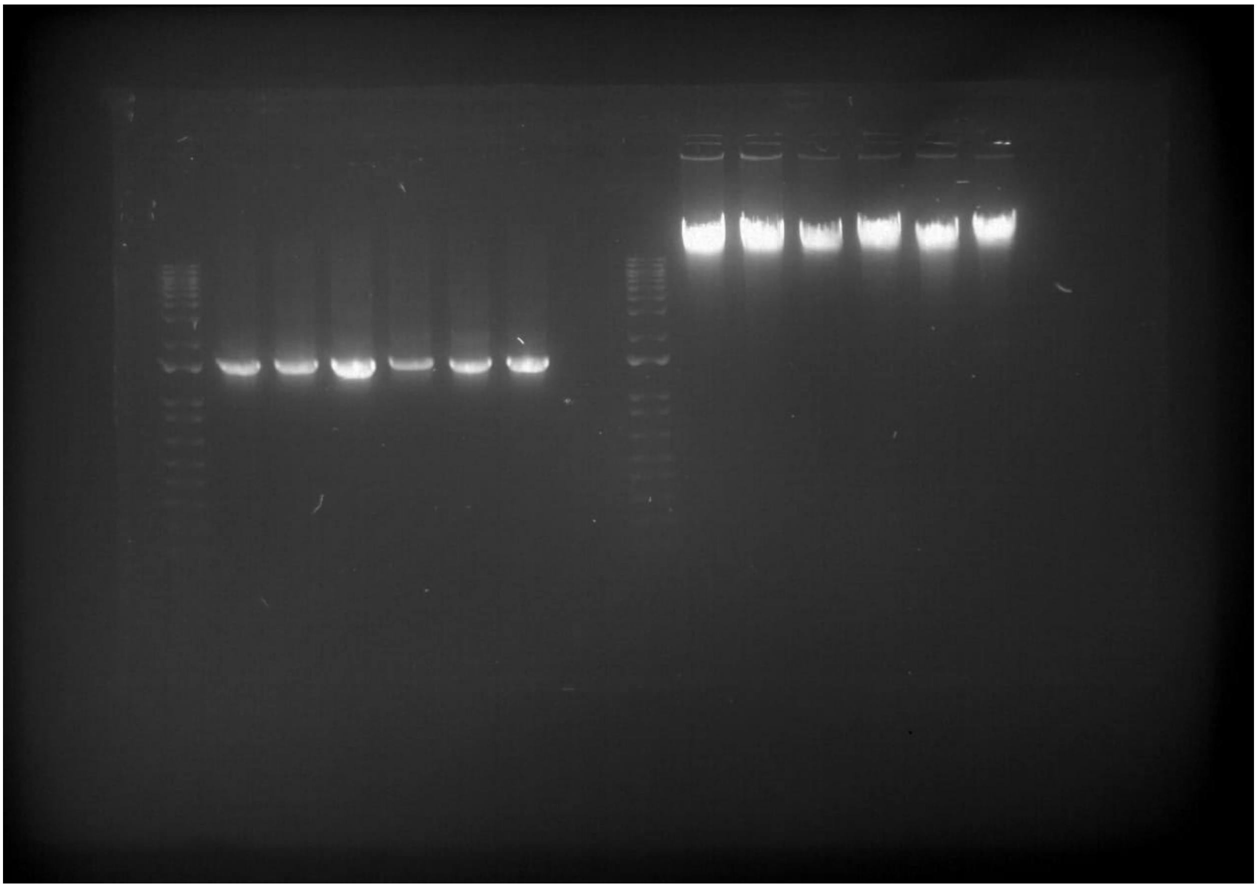

The lanes on the right correspond to additional samples that were not included in this study.
