# Supplementary material for: Isolation, Identification and Genomic Characterization of Carbamazepine-Tolerant Bacteria from Contaminated Aquatic Environments
Source: Microorganisms. 2026 Oct 10;14(10):2305. doi: 10.3390/microorganisms14102305 (PMC13707586; doi:10.3390/microorganisms14102305)
Supplement: Supplementary file 1 [file microorganisms-14-02305-s001.zip › Table S1 and S2.pdf]

Table S1: Biochemical reactions of strains under study.

	CBZ1	CBZ3	CBZ6	CBZ7
LYS	-	-	+	+
ORN	+	-	-	-
H2S	-	-	+	-
GLU	+	-	+	+
MAN	+	-	+	+
XYL	+	-	+	+
ONP	+	+	+	+
IND	-	-	+	+
URE	-	-	-	+
VP	+	-	+	-
CIT	+	-	+	+
TDA	-	-	+	-
GEL	+	+	+	+
MAL	+	-	-	+
INO	+	-	-	+
SOR	+	-	-	+
RHA	+	-	-	+
SUC	+	-	+	+
LAC	-	-	-	+
ARA	+	+	+	+
ADO	-	-	-	+
RAF	-	-	-	+
SAL	-	-	+	+
ARG	+	-	+	-

Note: LYS (Lysine), ORN (Ornithine), H2S (H2S production), GLU (Glucose), MAN (Mannitol), XYL (Xylose), ONP (ONPG), IND (Indole), URE (Urea), VP (Voges Proskauer), CIT (Citrate), TDA (Tryptophan, GEL (Gelatin Liquefaction), MAL (Malonate), INO (Inositol), SOR (Sorbitol), RHA (Rhamnose), SUC (Sucrose), LAC (Lactose), ARA (Arabinose), ADO (Adonitol), RAF (Raffinose), SAL (Salicin), ARG (Arginine).

Table S2. Molecular identification of carbamazepine-tolerant bacterial isolates based on 16S rRNA gene sequencing.

Strain	Organismo	% similarity	E-value	Query cover	NCBI sequence	Accession number
CBZ1	*Enterobacter hormaechei*	98.88%	0.0	100%	CP017180.1	PV916048
CBZ3	*Paenibacillus lactis*	99.40%	0.0	98.6%	AY257868	PV916049
CBZ6	*Aeromonas hydrophila*	96.57%	0.0	100%	CP000462	PV916050
CBZ7	*Raoultella ornithinolytica*	98.92%	0.0	100%	AJ251467	PV916051
